# Predicting inferior vena cava filter complications using machine learning

**DOI:** 10.1016/j.jvsv.2024.101943

**Published:** 2024-07-29

**Authors:** Ben Li, Naomi Eisenberg, Derek Beaton, Douglas S. Lee, Leen Al-Omran, Duminda N. Wijeysundera, Mohamad A. Hussain, Ori D. Rotstein, Charles de Mestral, Muhammad Mamdani, Graham Roche-Nagle, Mohammed Al-Omran

**Affiliations:** a Department of Surgery, University of Toronto, Toronto, Canada; b Division of Vascular Surgery, St. Michael’s Hospital, Unity Health Toronto, Toronto, Canada; c Institute of Medical Science, University of Toronto, Toronto, Canada; d Temerty Centre for Artificial Intelligence Research and Education in Medicine (T-CAIREM), University of Toronto, Toronto, Canada; e Division of Vascular Surgery, Peter Munk Cardiac Centre, University Health Network, Toronto, Canada; f Data Science & Advanced Analytics, Unity Health Toronto, University of Toronto, Toronto, Canada; g Division of Cardiology, Peter Munk Cardiac Centre, University Health Network, Toronto, Canada; h Institute of Health Policy, Management and Evaluation, University of Toronto, Toronto, Canada; i ICES, University of Toronto, Toronto, Canada; j School of Medicine, Alfaisal University, Riyadh, Saudi Arabia; k Department of Anesthesia, St. Michael’s Hospital, Unity Health Toronto, Toronto, Canada; l Li Ka Shing Knowledge Institute, St. Michael’s Hospital, Unity Health Toronto, Toronto, Canada; m Division of Vascular and Endovascular Surgery and the Center for Surgery and Public Health, Brigham and Women’s Hospital, Harvard Medical School, Boston, MA; n Division of General Surgery, St. Michael’s Hospital, Unity Health Toronto, Toronto, Canada; o Leslie Dan Faculty of Pharmacy, University of Toronto, Toronto, Canada; p Division of Vascular and Interventional Radiology, University Health Network, Toronto, Canada; q Department of Surgery, King Faisal Specialist Hospital and Research Center, Riyadh, Saudi Arabia

**Keywords:** Machine learning, Prediction, Complications, Inferior vena cava filter

## Abstract

**Objective:**

Inferior vena cava (*IVC*) filter placement is associated with important long-term complications. Predictive models for filter-related complications may help guide clinical decision-making but remain limited. We developed machine learning (*ML*) algorithms that predict 1-year IVC filter complications using preoperative data.

**Methods:**

The Vascular Quality Initiative database was used to identify patients who underwent IVC filter placement between 2013 and 2024. We identified 77 preoperative demographic and clinical features from the index hospitalization when the filter was placed. The primary outcome was 1-year filter-related complications (composite of filter thrombosis, migration, angulation, fracture, and embolization or fragmentation, vein perforation, new caval or iliac vein thrombosis, new pulmonary embolism, access site thrombosis, or failed retrieval). The data were divided into training (70%) and test (30%) sets. Six ML models were trained using preoperative features with 10-fold cross-validation (Extreme Gradient Boosting, random forest, Naïve Bayes classifier, support vector machine, artificial neural network, and logistic regression). The primary model evaluation metric was area under the receiver operating characteristic curve (*AUROC*). Model robustness was assessed using calibration plot and Brier score. Performance was evaluated across subgroups based on age, sex, race, ethnicity, rurality, median Area Deprivation Index, planned duration of filter, landing site of filter, and presence of prior IVC filter placement.

**Results:**

Overall, 14,476 patients underwent IVC filter placement and 584 (4.0%) experienced 1-year filter-related complications. Patients with a primary outcome were younger (59.3 ± 16.7 years vs 63.8 ± 16.0 years; *P* < .001) and more likely to have thrombotic risk factors including thrombophilia, prior venous thromboembolism (*VTE*), and family history of VTE. The best prediction model was Extreme Gradient Boosting, achieving an AUROC of 0.93 (95% confidence interval, 0.92-0.94). In comparison, logistic regression had an AUROC of 0.63 (95% confidence interval, 0.61-0.65). Calibration plot showed good agreement between predicted/observed event probabilities with a Brier score of 0.07. The top 10 predictors of 1-year filter-related complications were (1) thrombophilia, (2) prior VTE, (3) antiphospholipid antibodies, (4) factor V Leiden mutation, (5) family history of VTE, (6) planned duration of IVC filter (temporary), (7) unable to maintain therapeutic anticoagulation, (8) malignancy, (9) recent or active bleeding, and (10) age. Model performance remained robust across all subgroups.

**Conclusions:**

We developed ML models that can accurately predict 1-year IVC filter complications, performing better than logistic regression. These algorithms have potential to guide patient selection for filter placement, counselling, perioperative management, and follow-up to mitigate filter-related complications and improve outcomes.

**Article Highlights**

• **Type of Research:** Machine learning (ML)-based prognostic study using prospectively collected data from the Vascular Quality Initiative.
• **Key Findings:** ML models were trained on 14,476 patients undergoing inferior vena cava filter placement to predict 1-year filter-related complications, achieving an area under the receiver operating characteristic curve of 0.93 (95% confidence interval, 0.92-0.94) with good calibration using preoperative data.
• **Take Home Message:** ML models can accurately predict inferior vena cava filter complications and have potential to guide patient selection and periprocedural risk mitigation strategies.

Inferior vena cava (IVC) filters are generally placed in patients with deep vein thrombosis (DVT) to prevent pulmonary embolism (PE) when they have a contraindication to anticoagulation, an inability to achieve or maintain therapeutic levels of anticoagulation, or progression of PE despite anticoagulation, among other reasons.^1^ Given the significant morbidity and mortality associated with venous thromboembolism (VTE), which affects 1.2 million people in the United States and leads to mortality rates up to 35% within 1 year of diagnosis, IVC filters may play an important therapeutic role in select patients.^2^ However, IVC filter placement carries notable periprocedural and long-term risks, including rates of filter thrombosis, migration, and perforation rates up to 30%, 69%, and 24%, respectively.^3^ Therefore, accurate prediction of complications following IVC filter placement is critical to guide clinical decision-making, including patient selection for filter placement, counselling, and periprocedural management to mitigate adverse events.

There are currently no widely used tools to predict complications following IVC filter placement. The Society for Vascular Surgery (SVS) Vascular Quality Initiative (VQI) Cardiac Risk Index predicts outcomes after arterial, but not venous, interventions.^4^ Other tools such as the National Surgical Quality Improvement Program (NSQIP) online surgical risk calculator^5^ use modelling techniques that require manual input of clinical variables, which deters routine use in busy medical settings.^6^ With clinical judgment alone, the ability for clinicians to predict postprocedural complications is suboptimal, with previous studies demonstrating area under the receiver operating characteristic curve (AUROC) values ranging from 0.51 to 0.75.^7^ Therefore, there is an important need to develop more effective and practical tools to predict complications in patients being considered for IVC filter placement.

Machine learning (ML) is a rapidly advancing technology that allows computers to learn from data and make accurate predictions.^8^ Using advanced analytics, ML can model complex relationships between inputs (eg, patient characteristics) and outputs (eg, clinical outcomes).^8^ This field has been driven by the explosion of electronic information combined with increasing computational capabilities.^8^ The advantage of newer ML techniques over traditional statistical methods is that they can better model complex, multicollinear relationships between covariates and outcomes,^9^ which is common in health care data.^10^ Previously, ML was applied to the NSQIP database to develop an algorithm that predicts perioperative complications for >2900 distinct procedures.^11^ Given the heterogeneity of this cohort, better predictive performance may be achieved by building ML algorithms specific to patients undergoing IVC filter placement using the VQI database, a dedicated vascular registry containing procedure-specific variables.^12^ We previously described ML algorithms trained on VQI data for predicting outcomes after aortic, carotid, and peripheral arterial interventions, which achieved superior performance compared with traditional statistical techniques such logistic regression and existing tools.^13^, ^14^, ^15^, ^16^, ^17^, ^18^ The development of a ML-based risk prediction algorithm for IVC filter placement may complement these existing algorithms and expand clinical guidance for the management of patients with VTE. In this study, we used VQI data to develop ML algorithms that predict 1-year IVC filter complications using preoperative data. We hypothesized that our ML models could achieve better predictive performance compared with logistic regression.

## Methods

### Study approval

The SVS Patient Safety Organization Research Advisory Council approved this project and provided the deidentified dataset. Patient consent was not required as the data came from an anonymized registry.

### Design

This was a ML-based prognostic study and findings were reported based on the Transparent Reporting of a Multivariable Prediction Model for Individual Prognosis or Diagnosis + Artificial Intelligence statement.^19^

### Dataset

The VQI database is a clinical registry maintained by the SVS Patient Safety Organization with the goal of improving the delivery of vascular care (www.vqi.org).^12^ Vascular surgeons, interventionalists, and other specialists across >1000 academic and community hospitals in the United States, Canada, and Singapore prospectively contribute demographic, clinical, and outcomes data on consecutive eligible vascular patients, including information from their index procedure up to approximately 1 year of follow-up.^12^ Routine audits are performed to compare submitted data with hospital claims to ensure data accuracy.^20^

### Patient cohort

All patients who underwent IVC filter placement between January 1, 2013, and January 2, 2024, in the VQI database were included. IVC filters were placed by interventionalists or vascular surgeons through an endovascular approach for management of VTE in patients who had a contraindication to anticoagulation, could not maintain therapeutic levels of anticoagulation, or had recurrent PEs while on anticoagulation. Both temporary and permanent IVC filters were included. There were no exclusion criteria to maintain the generalizability of the cohort.

### Features

All predictor variables (features) used in the ML models were preoperative demographic and clinical patient characteristics. Given the advantage of ML in handling many input features, all available preoperative VQI variables were used to maximize predictive performance. There were 77 preoperative features including demographics (eg, age, sex, race, ethnicity, insurance status, and rurality), comorbidities (eg, smoking status, hypertension, diabetes, coronary artery disease [CAD], congestive heart failure [CHF], and chronic obstructive pulmonary disease), thrombotic risk factors (eg, thrombophilia, recent trauma, prior major amputation, prior VTE, family history of VTE, malignancy, and pregnancy), functional status (eg, living and ambulatory status), medications (eg, antiplatelets and anticoagulants), clinical presentation (eg, presence, severity, and location of DVT and/or PE, free floating thrombus, planned venous thrombolysis or thrombectomy, whether therapeutic anticoagulation could be provided, contraindications to anticoagulation, recurrent VTE while on anticoagulation, planned major procedures, and serum creatinine), and anatomical and IVC filter characteristics (eg, planned duration of filter, placement location, access vein, landing site, and abnormal venous anatomy). A complete list of preoperative features and their definitions can be found in Supplemental Table I.

### Outcome

The primary outcome was filter-related complications within 1 year after IVC filter placement. Filter-related complications were defined as a composite of filter thrombosis, migration, angulation, fracture, and embolization or fragmentation, vein perforation, new caval or iliac vein thrombosis, new PE, access site thrombosis, or failed retrieval. Filter thrombosis was defined as any amount of thrombus seen within the filter on imaging. Filter thrombosis included both partial and complete thrombosis; however, the VQI data dictionary does not differentiate between the two. Filter migration was defined as movement of the filter >20 mm cephalad or caudal from the original landing site. Filter angulation was defined as filter angle increase of >15° from initial placement based on center-line analysis of the IVC. Filter fracture was defined as a discontinuity of the filter seen on imaging. Filter embolization or fragmentation was defined as dislodgement of the filter causing the entire filter or a piece of the filter to be carried to a distant location in the systemic vasculature, such as the heart or pulmonary artery. Vein perforation was defined as transmural penetration of the venous vasculature by the IVC filter. New caval or iliac vein thrombosis was defined as new thrombosis of the caval or iliac veins that was not present before IVC filter placement. New PE was defined as a new PE that was not present before IVC filter placement. New PE was included in the composite outcome of filter-related complications because it may be related directly to manipulation of the venous system during filter placement or secondary to filter migration, angulation, fracture, or other complications during follow-up leading to a loss of efficacy of the filter in preventing a new PE.^21^ Access site thrombosis was defined as venous thrombosis at the access site used to place the IVC filter documented on imaging. Failed retrieval was defined as a failed attempt to retrieve the IVC filter. Failed retrieval was included in the composite outcome of filter-related complications because it may have occurred due to filter migration, fracture, embolization, fragmentation, or prolonged duration leading to increased risk of penetration of the filter hook, apex, or collar through the caval wall, leading to patient morbidity and mortality.^22^ Confirmation of filter complications was based on imaging studies ordered by the treating physician, including computed tomography angiography, magnetic resonance angiography, and/or conventional angiogram, among others. These imaging studies were ordered for routine follow-up of asymptomatic patients or to investigate symptoms and/or signs consistent with filter complications based on clinical assessment by the treating physician. These definitions were based on the VQI data dictionary.^12^ This composite outcome was chosen because it includes the most clinically relevant filter-related complications that can lead to major reinterventions and important morbidity and mortality, as previously described by other groups.^23^ Individual components of the primary outcome were not studied owing to the relatively low event rates of several individual outcomes (<0.1%), which would likely be inadequate for training an accurate predictive ML model.

### Model development

We trained six different ML models to predict 1-year IVC filter-related complications: Extreme Gradient Boosting (XGBoost), random forest, Naïve Bayes classifier, radial basis function support vector machine, multilayer perceptron artificial neural network, and logistic regression. These models were chosen based on their demonstrated accuracy in predicting postprocedural outcomes using structured data.^24^, ^25^, ^26^ Logistic regression was the baseline comparator because it is the most commonly applied statistical model in traditional risk prediction tools.^27^

The data were randomly divided into training (70%) and testing (30%) sets. Testing data were reserved for model evaluation and not used for training to ensure fair model evaluation. To determine the optimal model hyperparameters, 10-fold cross-validation and grid search were applied to training data.^28^^,^^29^ Initial analysis demonstrated that the primary outcome occurred in 584 of 14,476 patients (4.0%) in our cohort. To improve class balance, random over-sample examples (ROSE) was applied to training data.^30^ ROSE uses a smoothed bootstrapping approach to generate new samples from the feature space surrounding the minority class, a commonly used method to support predictive modelling of uncommon events.^30^ Given the application of ROSE, the probability of event cutoff used to indicate high risk of a primary outcome was 50%. The models were then evaluated on test set data and ranked based on the primary discriminatory metric of AUROC. Our best performing model was XGBoost, which had the following optimized hyperparameters: number of rounds = 200, maximum tree depth = 3, learning rate = 0.01, gamma = 0, column sample by tree = 1, minimum child weight = 1, and subsample = 0.9. Supplemental Table II outlines the process for selecting these hyperparameters.

### Statistical analysis

Preoperative demographic and clinical characteristics were summarized as means ± standard deviation or medians (interquartile range) for continuous variables and numbers (%) for categorical variables. Differences between patients with and without 1-year filter-related complications were assessed using independent t-tests (continuous variables) and χ^2^ tests (categorical variables). To account for multiple comparisons, Bonferroni correction was used to set statistical significance. The primary model evaluation metric was AUROC (95% confidence interval [CI]), a validated measure of discriminatory ability that considers both sensitivity and specificity.^31^ Secondary performance metrics were accuracy, sensitivity, specificity, positive predictive value, and negative predictive value. To assess model robustness, we plotted a calibration curve and calculated the Brier score, a measure of the agreement between predicted and observed event probabilities.^32^ In the final model, feature importance was determined by ranking the top 10 predictors based on variable importance scores (gain), a measure of the relative importance of individual covariates in contributing to an overall prediction.^33^ To assess model bias, we evaluated predictive performance across demographic/clinical subgroups based on age, sex, race, ethnicity, rurality, median Area Deprivation Index percentile, planned duration of filter, landing site of filter, and presence of prior IVC filter placement.

Based on a validated sample size calculator for clinical prediction models, to achieve a minimum AUROC of 0.8 with an outcome rate of approximately 4% and 77 preoperative features, a minimum sample size of 13,280 patients with 532 events is required.^34^ Our cohort of 14,476 patients with 584 primary events satisfied this sample size requirement. For variables of interest, missing data were <5%; hence, we adopted a complete-case analysis approach, considering only nonmissing covariates for each patient. This is a valid analytical method for datasets with minimal missing data (<5%) and reflects predictive modelling of real-world data, which inherently includes missing information.^35^^,^^36^ Patients lost to follow-up were censored. Owing to the relatively low percentage of patients lost to follow-up (<5%), individuals without documented 1-year follow-up were placed in the complication-free category. Patients were not excluded due to missing data to reduce the risk of selection bias. All analyses were conducted using R version 4.3.1.^37^

## Results

### Patients, events, and follow-up

A total of 14,476 patients underwent IVC filter placement in the VQI database between January 1, 2013, and January 2, 2024. Sixty-four sites contributed data to this study and the median number of patients contributed per site was 140 (IQR, 82-263). The maximum number of patients contributed by a single site was 1093 (7.6%), and the remainder contributed <1000 patients, suggesting that the overall cohort was not heavily influenced by a single or small number of contributing sites. In terms of access veins, 5263 filters (36.4%) were placed through a transjugular approach, and 8991 filters (62.1%) were placed via a transfemoral approach, while the remainder were placed through a nonfemoral lower extremity vein, or the access vein was not reported. Overall, 584 (4.0%) experienced 1-year filter-related complications, including filter thrombosis (n = 140 [1.0%]), migration (n = 12 [0.08%]), angulation >15° (n = 62 [0.4%]), fracture (n = 6 [0.04%]), embolization or fragmentation (n = 2 [0.01%]), vein perforation (n = 48 [0.3%]), new caval or iliac vein thrombosis (n = 75 [0.5%]), new PE (n = 112 [0.8%]), access site thrombosis (n = 56 [0.4%]), and failed retrieval (n = 205 [1.4%]). The mean and median follow-up times were 13.3 ± 1.2 months and 13.4 months (IQR, 12.2-15.4 months) in both the training and test sets, respectively. A follow-up visit was documented for 14,194 (98.1%) at 1 year after IVC filter placement and 5363 filters (37.0%) were removed within 1 year of placement. After IVC filter placement, 7016 patients (48.5%) received anticoagulation before discharge from their index hospitalization for filter placement. The specific timepoint at which anticoagulation was started for individual patients after filter placement was not reported in the dataset.

### Preoperative characteristics

Compared to patients without a primary outcome, those who developed 1-year filter-related complications were younger (59.3 ± 16.7 years vs 63.8 ± 16.0 years; *P* < .001) and had a higher mean body mass index (31.9 ± 9.8 vs 30.8 ± 9.4; *P* = .005), with no differences in sex, race, ethnicity, insurance status, and rurality of residence between groups. Patients with 1-year filter-related complications were less likely to have hypertension, diabetes, CAD, or CHF. A greater proportion of patients with an adverse outcome had thrombophilia, particularly antiphospholipid antibodies, Factor V Leiden mutation, prothrombin 20210A mutation, and antithrombin deficiency. They were also more likely to have other thrombotic risk factors including a prior VTE and a family history of VTE, but less likely to be diagnosed with an active malignancy. Functionally, patients with 1-year filter-related complications were more likely to live at home and ambulate independently. There were no differences between the groups in terms of medications received, including oral and intravenous or subcutaneous anticoagulation. For clinical presentation, the rate, severity, and location of DVT and PE were similar between groups, whereas patients with an adverse outcome were more likely to be unable to maintain a therapeutic level of anticoagulation and have planned venous thrombolysis or thrombectomy. They were also more likely to have planned major surgery, suffer recurrent VTE while on anticoagulation, and receive a temporary filter (Table I).

**Table I:** Preoperative demographic and clinical characteristics of patients undergoing inferior vena cava (IVC) filter placement with and without 1-year filter-related complications

	Absence of 1-year filter-related complications (n = 13,892)	Presence of 1-year filter-related complications (n = 584)	*P* value
Demographics			
Age, years	63.8 ± 16.0	59.3 ± 16.7	<.001
Female	6658 (47.9)	286 (49.0)	.65
BMI, kg/m^2^	30.8 ± 9.4	31.9 ± 9.8	.005
Race			
American Indian or Alaskan Native	46 (0.3)	3 (0.5)	.84
Asian	250 (1.8)	8 (1.4)	
Black	2746 (19.8)	116 (19.9)	
Native Hawaiian or other Pacific Islander	15 (0.1)	1 (0.2)	
White	10,021 (72.1)	424 (72.6)	
>1 race	35 (0.3)	3 (0.5)	
Unknown/other	779 (5.6)	29 (5.0)	
Hispanic ethnicity	526 (3.8)	19 (3.3)	.58
Insurance status			
Medicare	6314 (45.5)	226 (38.7)	.07
Medicaid	1101 (7.9)	48 (8.2)	
Commercial	5701 (41.0)	273 (46.7)	
Military/Veterans Affairs	186 (1.3)	6 (1.0)	
Non-US Insurance	21 (0.2)	1 (0.2)	
Self-pay (uninsured)	553 (4.0)	30 (5.1)	
Unknown/other	16 (0.1)	0	
Rural residence	477 (3.4)	28 (4.8)	.10
Area Deprivation Index percentile	46 (21-73)	45 (16-72)	.03
Transfer status			
From another hospital	2558 (18.4)	114 (19.5)	.69
From rehabilitation unit	416 (3.0)	15 (2.6)	
Comorbidities			
Smoking status			
Never	7485 (53.9)	316 (54.1)	.82
Prior	4443 (32.0)	181 (31.0)	
Current	1964 (14.1)	87 (14.9)	
Hypertension	9099 (65.5)	347 (59.4)	.001
Diabetes	3599 (25.9)	116 (19.9)	.01
CAD	1581 (11.4)	34 (5.8)	.002
CHF	1573 (11.3)	41 (7.0)	.02
Chronic obstructive pulmonary disease			
Not treated	433 (3.1)	12 (2.1)	.15
On medications	1465 (10.5)	51 (8.7)	
On home oxygen	413 (3.0)	14 (2.4)	
Dialysis	343 (2.5)	6 (1.0)	.08
Thrombotic risk factors			
Thrombophilia	935 (6.7)	63 (10.8)	<.001
Antiphospholipid antibodies	55 (0.4)	12 (2.1)	<.001
Excess factor VIII	19 (0.1)	0	.76
Excess factor XI	4 (0.03)	0	.99
Factor V Leiden mutation	271 (2.0)	26 (4.5)	<.001
Hyperhomocysteinemia	14 (0.1)	2 (0.3)	.28
Protein C deficiency	33 (0.2)	4 (0.7)	.09
Protein S deficiency	44 (0.3)	3 (0.5)	.65
Prothrombin 20210A mutation	39 (0.3)	6 (1.0)	.005
Antithrombin deficiency	15 (0.1)	5 (0.9)	<.001
Other thrombophilia	449 (3.2)	16 (2.7)	.59
Recent trauma within last 30 days	619 (4.5)	20 (3.4)	.28
Head	551 (4.0)	23 (3.9)	.99
Long bones	385 (2.8)	17 (2.9)	.94
Solid organ	129 (0.9)	7 (1.2)	.66
Spine	303 (2.2)	12 (2.1)	.95
Other trauma	345 (2.5)	8 (1.4)	.12
Prior major amputation			
Below or through knee	72 (0.5)	1 (0.2)	.23
Above knee	50 (0.4)	4 (0.7)	
Prior VTE			
Yes, with no prior IVC filter	4258 (30.7)	219 (37.5)	<.001
Yes, with prior IVC filter	267 (1.9)	19 (3.3)	
Family history of VTE	522 (3.8)	39 (6.7)	<.001
Malignancy			
Cured or in remission	988 (7.1)	48 (8.2)	<.001
Active	3604 (25.9)	110 (18.8)	
Pregnancy			
Delivered >30 days ago	3442 (24.8)	143 (24.5)	.93
Delivered within last 30 days	32 (0.2)	2 (0.3)	
Current	35 (0.3)	1 (0.2)	
Functional status			
Living status			
Home	12,771 (91.9)	557 (95.4)	.004
Nursing home	1049 (7.6)	23 (3.9)	
Homeless	48 (0.3)	4 (0.7)	
Not reported	24 (0.2)	0	
Ambulatory status			
Ambulatory independently with or without prosthesis	9756 (70.2)	448 (76.7)	.005
Ambulatory with assistance (eg, cane, walker, or person)	2849 (20.5)	102 (17.5)	
Wheelchair dependent	656 (4.7)	22 (3.8)	
Bedridden	596 (4.3)	11 (1.9)	
Not reported	35 (0.3)	1 (0.2)	
Medications			
Acetylsalicylic acid	3060 (22.0)	111 (19.0)	.21
P2Y12 antagonist	497 (3.6)	16 (2.7)	.84
Statin	4234 (30.5)	165 (28.3)	.47
Oral anticoagulant	2107 (15.2)	104 (17.8)	.46
Intravenous or subcutaneous anticoagulant	5096 (36.7)	228 (39.0)	.43
Estrogen-containing therapy	258 (1.9)	15 (2.6)	.28
Clinical presentation			
PE			
Asymptomatic	1063 (7.7)	44 (7.5)	.15
Mild symptoms	2263 (16.3)	107 (18.3)	
Severe symptoms	1013 (7.3)	44 (7.5)	
Massive, treated with lysis or thrombectomy	801 (5.8)	42 (7.2)	
Chronic treated with thrombectomy	13 (0.09)	2 (0.3)	
Lower extremity DVT			
Right	3161 (22.8)	128 (21.9)	.88
Left	3612 (26.0)	152 (26.0)	
Bilateral	2089 (15.0)	84 (14.4)	
DVT location on right leg			
Soleal/gastrocnemius vein	420 (3.0)	11 (1.9)	.08
Peroneal vein	309 (2.2)	11 (1.9)	
Tibial vein	457 (3.3)	27 (4.6)	
Popliteal vein	998 (7.2)	49 (8.4)	
Femoral vein	1434 (10.3)	48 (8.2)	
Common femoral vein	1129 (8.1)	42 (7.2)	
External iliac vein	255 (1.8)	6 (1.0)	
Common iliac vein	101 (0.7)	6 (1.0)	
IVC	74 (0.5)	6 (1.0)	
DVT location on left leg			
Soleal/gastrocnemius vein	421 (3.0)	14 (2.4)	.63
Peroneal vein	295 (2.1)	12 (2.1)	
Tibial vein	454 (3.3)	22 (3.8)	
Popliteal vein	1037 (7.5)	46 (7.9)	
Femoral vein	1550 (11.2)	60 (10.3)	
Common femoral vein	1242 (8.9)	50 (8.6)	
External iliac vein	320 (2.3)	10 (1.7)	
Common iliac vein	204 (1.5)	8 (1.4)	
IVC	90 (0.6)	8 (1.4)	
Free-floating thrombus	305 (2.2)	16 (2.7)	.47
Planned venous thrombolysis or thrombectomy	424 (3.1)	27 (4.6)	.04
Anticoagulation at therapeutic target			
Yes	2195 (15.8)	109 (18.7)	.001
No, contraindicated	7725 (55.6)	281 (48.1)	
No, unable to maintain therapeutic level	962 (6.9)	56 (9.6)	
Not reported	3010 (21.7)	138 (23.6)	
Contraindications for anticoagulation			
High risk of fall or injury	696 (5.0)	16 (2.7)	.02
Heparin-induced thrombocytopenia	65 (0.5)	2 (0.3)	.90
Nonbleeding complications (ie, skin necrosis, allergy)	50 (0.4)	2 (0.3)	.99
Planned major surgery	1290 (9.3)	70 (12.0)	.03
Recent cerebrovascular event (ie, intracranial bleed or stroke)	1159 (8.3)	50 (8.6)	.91
Recent major surgery	1233 (8.9)	54 (9.3)	.81
Recent trauma	544 (3.9)	22 (3.8)	.94
Recent or active bleeding	4460 (32.1)	130 (22.3)	<.001
Other	698 (5.0)	17 (2.9)	.03
Recurrent VTE on anticoagulation			
New or extension DVT	483 (3.5)	29 (5.0)	.03
New PE	343 (2.5)	21 (3.6)	
Major procedure planned			
Bariatric	372 (2.7)	17 (2.9)	.22
Orthopedic	718 (5.2)	32 (5.5)	
Central nervous system	478 (3.4)	16 (2.7)	
Chest or abdomen	346 (2.5)	24 (4.1)	
Other	262 (1.9)	12 (2.1)	
Creatinine, μmol/L	91.3 ± 64.1	85.0 ± 47.1	.02
Anatomical and IVC filter characteristics			
Planned duration of IVC filter			
Temporary	11,607 (83.6)	549 (94.0)	<.001
Permanent	2235 (16.1)	34 (5.8)	
Not reported	50 (0.4)	1 (0.2)	
IVC filter placement location			
Fluoroscopy suite	13,620 (98.0)	573 (98.1)	.58
Bedside	247 (1.8)	11 (1.9)	
Not reported	25 (0.2)	0	
Access vein			
Right jugular vein	4998 (36.0)	180 (30.8)	.004
Left jugular vein	83 (0.6)	2 (0.3)	
Right femoral vein	7379 (53.1)	343 (58.7)	
Left femoral vein	1220 (8.8)	49 (8.4)	
Right leg nonfemoral vein	114 (0.8)	4 (0.7)	
Left leg nonfemoral vein	43 (0.3)	6 (1.0)	
Not reported	55 (0.4)	0	
Landing site			
Infrarenal IVC	13,358 (96.2)	561 (96.1)	.15
Pararenal IVC	265 (1.9)	5 (0.9)	
Suprarenal IVC	179 (1.3)	12 (2.1)	
Right iliac vein	16 (0.1)	2 (0.3)	
Left iliac vein	13 (0.1)	1 (0.2)	
Bilateral iliac veins	9 (0.1)	1 (0.2)	
Not reported	52 (0.4)	2 (0.3)	
Imaging available to place filter			
Fluoroscopy	10,956 (78.9)	495 (84.8)	.003
Transcutaneous ultrasound	2659 (19.1)	77 (13.2)	
Intravascular ultrasound	256 (1.8)	12 (2.1)	
Not reported	21 (0.2)	0	
Abnormal venous anatomy (ie, IVC compression, tortuosity, or duplication, accessory renal vein, or large low lying gonadal vein)	236 (1.7)	13 (2.2)	.43

*BMI*, Body mass index; *CAD*, coronary artery disease; *CHF*, congestive heart failure; *DVT*, deep vein thrombosis; *PE*, pulmonary embolism; *VTE*, venous thromboembolism.

Values are reported as number (%), mean ± standard deviation, or median (interquartile range).

### Model performance

**Fig 1:**
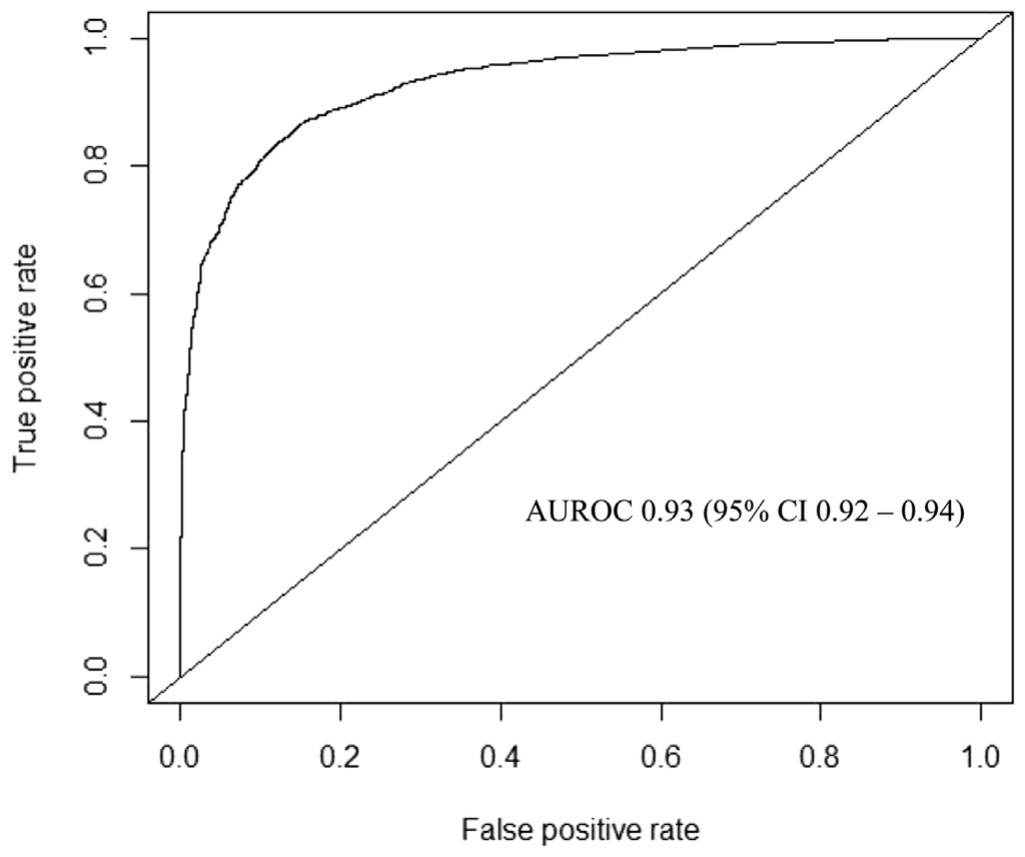
Receiver operating characteristic curve for predicting 1-year filter-related complications after inferior vena cava (IVC) filter placement using preoperative data with Extreme Gradient Boosting (XGBoost) model. *AUROC*, area under the receiver operating characteristic curve; *CI*, confidence interval.

**Fig 2:**
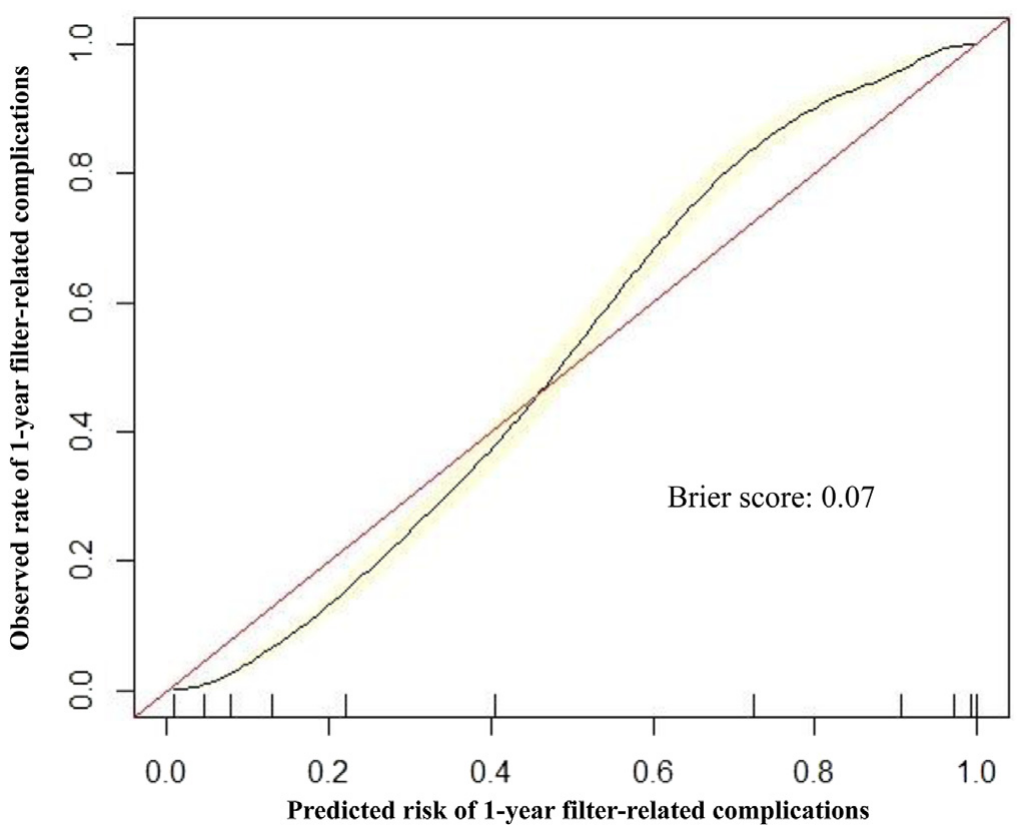
Calibration plot with Brier score for predicting 1-year filter-related complications following inferior vena cava (IVC) filter placement using Extreme Gradient Boosting (XGBoost) model.

**Fig 3:**
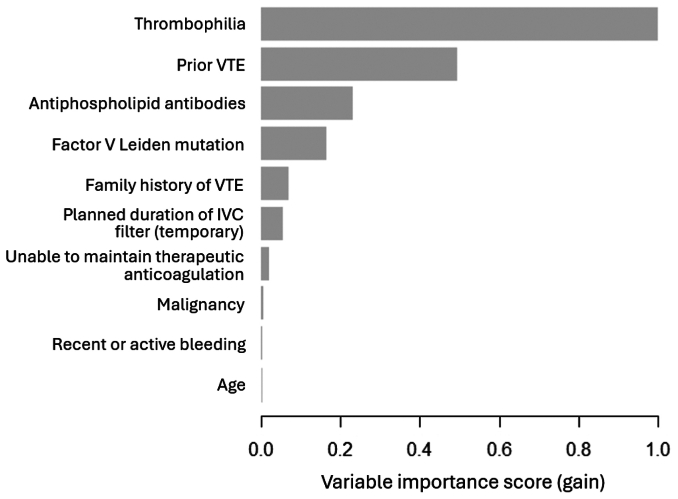
Variable importance scores (gain) for the top 10 predictors of 1-year filter-related complications after IVC filter placement in the Extreme Gradient Boosting (XGBoost) model. *IVC*, inferior vena cava; *VTE*, venous thromboembolism.

Of the six ML models evaluated using test set data, XGBoost had the best performance in predicting 1-year filter-related complications (AUROC, 0.93; 95% CI, 0.92–0.94) (Fig 1). In comparison, the other models had the following AUROCs: random forest (0.92; 95% CI, 0.91-0.93), Naïve Bayes (0.86; 95% CI, 0.85-0.88), radial basis function support vector machine (0.84; 95% CI, 0.82-0.85), multilayer perceptron artificial neural network (0.78; 95% CI, 0.76-0.80), and logistic regression (0.63; 95% CI, 0.61-0.65). The secondary performance metrics of XGBoost were the following: accuracy 0.85 (95% CI, 0.84-0.86), sensitivity 0.85, specificity 0.86, positive predictive value 0.83, and negative predictive value 0.87. Model performance results are summarized in Table II. There was good agreement between predicted and observed event probabilities as demonstrated by the calibration plot in Fig 2, with a Brier score of 0.07. The top 10 predictors of 1-year filter-related complications in the final XGBoost model were (1) thrombophilia, (2) prior VTE, (3) antiphospholipid antibodies, (4) factor V Leiden mutation, (5) family history of VTE, (6) planned duration of IVC filter (temporary), (7) unable to maintain therapeutic anticoagulation, (8) malignancy, (9) recent or active bleeding, and (10) age (Fig 3).

**Table II:** Model performance on test set data for predicting 1-year filter-related complications following inferior vena cava (IVC) filter placement using preoperative features

	AUROC (95% CI)	Accuracy (95% CI)	Sensitivity	Specificity	PPV	NPV
XGBoost	0.93 (0.92-0.94)	0.85 (0.84-0.86)	0.85	0.86	0.83	0.87
Random forest	0.92 (0.91-0.93)	0.84 (0.82-0.85)	0.83	0.84	0.85	0.82
Naïve Bayes	0.86 (0.85-0.88)	0.80 (0.79-0.81)	0.79	0.81	0.81	0.78
RBF SVM	0.84 (0.82-0.85)	0.76 (0.74-0.77)	0.78	0.74	0.72	0.80
MLP ANN	0.78 (0.76-0.80)	0.75 (0.74-0.77)	0.76	0.75	0.73	0.78
Logistic regression	0.63 (0.61-0.65)	0.54 (0.53-0.56)	0.53	0.62	0.58	0.50

*AUROC*, Area under the receiver operating characteristic curve; *CI*, confidence interval; *MLP ANN*, multilayer perceptron artificial neural network; *NPV*, negative predictive value; *PPV*, positive predictive value; *RBF SVM*, radial basis function support vector machine; *XGBoost*, Extreme Gradient Boosting.

### Subgroup analysis

Model performance remained robust on subgroup analyses across demographic/clinical populations based on age, sex, race, ethnicity, rurality, median Area Deprivation Index percentile, planned duration of filter, landing site of filter, and presence of prior IVC filter placement with AUROCs ranging from 0.92 to 0.94 and no significant differences between majority and minority groups (Supplemental Figs 1-9).

## Discussion

### Summary of findings

We used data from a large clinical registry consisting of 14,476 patients who underwent IVC filter placement to develop ML models that accurately predict 1-year postprocedural filter-related complications using preoperative data with an AUROC of 0.93. There were several key findings. First, patients who suffer filter-related complications have several preoperative predictive features related to their thrombotic risk factors, comorbidities, clinical presentation, and anatomical and IVC filter characteristics. ML-based modeling allowed us to assess the combined impact of these factors on the risk of complications. Second, we trained and evaluated six ML models on our dataset and XGBoost achieved the best performance, demonstrating excellent discrimination and calibration. Furthermore, predictive performance remained robust across demographic and clinical subpopulations. Third, we identified the top 10 predictive features in the ML model, which primarily included thrombotic risk factors, including thrombophilia, prior VTE, and family history of VTE. These features provide clinical explainability for our model and can guide clinicians in terms of individualized risk assessment, patient selection for intervention, and perioperative management.

### Comparison with the existing literature

IVC filter complications have been previously characterized, but there are no existing validated models to predict the risk of filter-related complications.^23^ Ramakrishnan et al (2023) recently evaluated IVC filter complications using VQI data from 2013 to 2020.^23^ The authors found a delayed filter-related complication rate of 3.1%, including filter migration, angulation, fracture, thrombosis, fragmentation or embolization, caval and/or iliac thrombosis, and vein perforation.^23^ Using a more updated VQI cohort, we similarly demonstrated a 1-year filter-related complication rate of 4.0%.^23^ Therefore, complications after IVC filter placement are non-negligible; they may require technically challenging reinterventions and lead to morbidity or mortality or both.^23^ The authors demonstrated that important risk factors for delayed complications included a family history of VTE and imaging available to place the filter (ie, intravascular ultrasound and/or fluoroscopy).^23^ We similarly demonstrated that these variables were significantly different between patients with and without 1-year filter-related complications, with a family history of VTE being a top five predictive feature in our ML model. Ramakrishnan et al^23^ were interested primarily in characterizing the incidence of IVC filter complications and secondarily assessing the risk factors for complications. In the present study, we expanded the use of this VQI registry and built an accurate ML-based predictive model for 1-year IVC filter complications using preoperative features, which may provide greater utility in the routine clinical setting to guide decision-making.

Bonde et al.^11^ trained ML algorithms on a cohort of patients who underwent >2900 unique procedures in the NSQIP database to predict perioperative complications, achieving AUROCs between 0.85 and 0.88.^11^ Given that patients being considered for IVC filter placement are generally a high-risk group characterized by unique comorbidities and clinical presentations that generally predispose them to a prothrombotic state and may be unable to receive therapeutic anticoagulation, the usefulness of generic risk prediction tools may have limitations.^38^ By developing ML algorithms tailored to patients undergoing IVC filter placement, we achieved an AUROC of >0.90. Furthermore, our model has been trained to predict filter-specific complications, including filter thrombosis, migration, fracture, and angulation, among others, which are of clinical importance to interventionalists and vascular surgeons.^39^ Therefore, we demonstrate the value of building procedure-specific ML models, which can improve performance and clinical applicability. This prediction model for IVC filter complications complements our previously described ML algorithms for predicting outcomes after arterial interventions, which achieved similarly excellent performance with AUROCs of ≥0.90.^13^, ^14^, ^15^, ^16^, ^17^, ^18^

### Explanation of findings

There are several explanations for our findings. First, patients who suffer IVC filter complications represent a unique population with multiple risk factors, which is corroborated by the previous literature.^40^ In particular, we demonstrated that thrombotic risk factors, including thrombophilia, prior VTE, antiphospholipid antibodies, Factor V Leiden mutation, and family history of VTE were the top five predictors of 1-year filter-related complications in our ML models. This is corroborated by previous literature and suggests that patients in a highly prothrombotic state are likely to experience complications including filter thrombosis, migration, and angulation, as well as new VTE despite filter placement.^39^ Paradoxically, we found that the absence of comorbidities including hypertension, diabetes, CAD, CHF, and active malignancy were associated with a higher rate of 1-year filter-related complications. One potential explanation for this finding is that patients with fewer comorbidities are less likely to receive regular health care and follow-up.^41^ Sadri et al.^41^ demonstrated that only 22% of patients who received retrievable IVC filters presented for filter retrieval, and a significant proportion of patients were lost to follow-up. Given that IVC filters that remain in situ for prolonged periods when they are no longer necessary can increase the risk of complications, patients who do not interact regularly with the health care system may be more easily lost to follow-up and, therefore, suffer filter-related complications in the long term.^41^ We demonstrated that although 84% of patients received temporary filters, only 37% of individuals had their filters retrieved within 1 year of placement. This finding suggests a relatively high indwelling time for temporary filters, which has been demonstrated to be directly related to complications, such as limb fracture.^39^ Therefore, it is important for clinicians to identify this population of patients with few comorbidities who are at risk of loss-to-follow-up and ensure that a clear follow-up protocol is established for filter monitoring and retrieval to decrease the risk of adverse events.^42^ Second, we demonstrated that preoperative features can accurately predict 1-year filter-related complications. This finding suggests that the ability to predict long-term complications after IVC filter placement accurately can be established preprocedurally to support decision-making regarding patient selection, counselling, periprocedural management, and follow-up. Third, our ML models performed better than existing tools for several potential reasons. Compared with traditional logistic regression, advanced ML techniques can better model the complex, nonlinear relationships between inputs and outputs.^43^ This is especially important in health care data, because patient outcomes can be influenced by many demographic, clinical, and system-level factors.^44^ Our top-performing algorithm was XGBoost, which has unique advantages, including relatively fewer issues with overfitting and faster computing while maintaining precision.^45^, ^46^, ^47^ Furthermore, XGBoost works well with structured data, which may explain its superior performance compared with more complex algorithms, such as neural networks, on our dataset.^48^ Fourth, the performance of our models remained robust on subgroup analyses of specific demographic and clinical populations. This finding is important given that algorithm bias against under-represented populations is a frequently encountered issue in ML models.^49^ We were likely able to avoid such biases owing to the excellent capture of sociodemographic data in the VQI.^12^

### Implications

Our ML models can help to guide clinical decision-making in several ways for patients being considered for IVC filter placement. Using preoperative data, an individual predicted to be at high risk of complications should be assessed further in terms of modifiable and nonmodifiable factors.^50^ Patients with significant nonmodifiable risks may benefit from alternative management options, including a reassessment of their anticoagulation strategy and consideration of thrombolysis or thrombectomy after multidisciplinary discussion with hematologists, respirologists, interventionalists, and vascular surgeons, as well as other team members.^51^^,^^52^ Patients with modifiable risks, such as untreated underlying thrombotic disorders, may benefit from further evaluation and optimization with appropriate referrals to medical specialists, including hematologists and thrombosis specialists.^53^ Postoperatively, patients flagged as being at high risk for adverse events such as filter fragmentation or embolization, vein perforation, and/or recurrent PE may be monitored closely in the intensive care unit to provide timely intervention if complications arise.^54^ Furthermore, high-risk patients may benefit from early support from a multidisciplinary team to optimize safe discharge planning with establishment of a clear follow-up plan for filter monitoring and retrieval.^42^^,^^51^ These perioperative decisions guided by our tool have the potential to improve patient-centered care by predicting and potentially helping to mitigate complications after IVC filter placement to improve outcomes.

The programming code used to develop our ML models is available publicly on GitHub, allowing clinicians involved in the periprocedural management of patients being considered for IVC filter placement to use our tool. At a system-wide level, our models can be implemented by the >1000 centers that participate in VQI.^12^ The VQI database managers at these institutions routinely capture the input features used in our ML algorithms.^12^ The number of VQI centers has grown considerably from 400 in 2019 to >1000 in 2024.^12^^,^^55^ Recently, the VQI recorded >1 million procedures.^56^ Therefore, our models have broad and growing utility. They also have potential for use beyond VQI sites, because the predictors for our models are commonly captured variables for the routine care of patients with VTE being considered for IVC filter placement.^57^ Given the challenges of deploying prediction models into practice, thoughtful consideration of implementation science principles is critical.^58^ A key advantage of our ML models is their ability to provide automated risk predictions, thereby enhancing feasibility in busy clinical settings compared with traditional risk predictors that often require manual input of variables.^5^ Specifically, our ML algorithms can extract a patient's VQI data autonomously to generate risk predictions. To facilitate successful implementation of our ML tool, we recommend establishing and supporting data analytics teams at the institutional level. Such teams can provide important benefits to patient care, and their expertise can facilitate the deployment of our ML models.^59^ Without access to the full model, a clinician may assess the number of risk factors from the top 10 predictive features for filter-related complications identified in this study to provide a rough estimate of a patient's potential risk for complications. However, to make full use of the ML algorithm and obtain an accurate risk prediction, we recommend running the model in real time with the support of a data analytics team using all available patient information.

### Limitations

Our study has several limitations. First, our models were developed with VQI data, a voluntary registry primarily comprising data from North American centers. Future studies are needed to assess whether performance can be generalized beyond VQI sites. Second, although we evaluated six different ML models, there are other ML models available.^60^ We chose these six models because of their established efficacy for predicting postprocedural complications using structured data.^24^ We achieved excellent performance; however, ongoing evaluation of novel ML techniques would be prudent. Third, our models only included preoperative variables, because this infromation provides the most opportunity to guide clinical decision-making, such as patient selection for filter placement. Intraoperative and immediate postoperative variables for IVC filter placement were not well-captured in our dataset, including the size and type of filter placed, technical complications, and in-hospital postoperative course. Training of future models using more extensive intraoperative and postoperative data may further enhance model performance for predicting long-term filter-related complications. Fourth, the development of a prediction model specifically for failed retrieval may be helpful in guiding decision-making regarding filter placement. However, the event rate for failed retrieval in our dataset was too low (<2%) to support the development of an accurate prediction model. As IVC filter data accumulate in the VQI database, there may be opportunities to develop a model specifically to predict failed retrieval in future studies. Fifth, our dataset was blinded to filter type. Given that various filter types have different designs and potentially variable complication rates, future ML models developed using datasets that include filter type as an input feature may improve predictive performance.^61^

## Conclusions

We used a large, vascular-specific clinical registry (VQI) to develop a robust ML model that predicts 1-year IVC filter complications using preoperative data with excellent performance (AUROC 0.93). Our model can support individualized risk assessment and guide patient selection, counselling, perioperative management, and follow-up care to prevent filter-related complications. Notably, our model remained robust across demographic/clinical subpopulations and outperformed existing prediction tools and logistic regression, and, therefore, has potential for important utility in the care of patients being considered for IVC filter placement. Prospective validation of our ML algorithm is warranted.

## Code availability statement

The complete code used for model development and evaluation in this project is publicly available on GitHub: https://github.com/benli12345/IVC-ML-VQI.

## Data availability statement

The data used for this study comes from the VQI, which is maintained by the Society for Vascular Surgery Patient Safety Organization. Access and use of the data requires approval through an application process available at https://www.vqi.org/data-analysis/.

## Author Contributions

Conception and design: BL, NE, DB, CM, MM, GR, MA

Analysis and interpretation: BL, NE, DB, DL, LA, DW, MH, OR, CM, MM, GR, MA

Data collection: BL, NE

Writing the article: BL

Critical revision of the article: BL, NE, DB, DL, LA, DW, MH, OR, CM, MM, GR, MA

Final approval of the article: BL, NE, DB, DL, LA, DW, MH, OR, CM, MM, GR, MA

Statistical analysis: BL, DB

Obtained funding: BL

Overall responsibility: MA

## Declaration of generative AI and AI-assisted technologies in the writing process

Generative AI and AI-assisted technologies were not used in the writing process.

## Funding

This research was partially funded by the 10.13039/501100000024Canadian Institutes of Health Research, 10.13039/501100025092Ontario Ministry of Health, 10.13039/501100000241PSI Foundation, and Schwartz Reisman Institute for Technology and Society at the University of Toronto (B.L.). The funding sources did not play a role in study design, collection, analysis, or interpretation of data, manuscript writing, creation of the manuscript, or the decision to submit the manuscript for publication.

## Disclosures

None.
