# Supplementary material for: Predicting inferior vena cava filter complications using machine learning
Source: J Vasc Surg Venous Lymphat Disord. 2024 Jul 29;12(6):101943. doi: 10.1016/j.jvsv.2024.101943 (PMC11523346; doi:10.1016/j.jvsv.2024.101943)
Supplement: Supplemental Tables and Figures [file mmc1.docx]

**Supplementary Table 1. Pre-operative features for machine learning models**

| **Features (n = 77)** | **Definition based on Vascular Quality Initiative Data Dictionary** |
| --- | --- |
| **Logistics** |  |
| Region | Region of procedure (deidentified) |
| Center | Center of procedure (deidentified) |
| Physician | Physician who performed procedure (deidentified) |
| Intervention year | Year of intervention |
| Intervention month | Month of intervention |
| Intervention weekday | Day of week that intervention was performed |
| **Demographics** |  |
| Age | Age in years |
| Sex | Male or female |
| Body mass index | Weight in kg / height in m^2^ |
| Race | Patient-reported race including White, Black, Asian, American Indian or Alaskan Native, Native Hawaiian or other Pacific Islander, more than 1 race, or unknown/other |
| Ethnicity | Hispanic or non-Hispanic |
| Primary insurer | Primary method of health insurance including Medicare, Medicaid, Commercial, Medicare Advantage, Military or Veterans Affairs, Non-US Insurance, self-pay (uninsured), or unknown/other |
| Rural residence | Defined based on the patient’s primary rural-urban commuting area (RUCA) code based on the most recent publicly available dataset. Rural residence is RUCA code 10 (<https://www.ers.usda.gov/data-products/rural-urban-commuting-area-codes/documentation//>). |
| Median Area Deprivation Index (ADI) | National percentile rank based on the most recent publicly available dataset. Calculated by taking the median ADI national percentile rank among all 9-digit zip code records that contain the patient’s 5-digit zip code prefix (<https://www.neighborhoodatlas.medicine.wisc.edu/>). A higher number indicates a greater level of socioeconomic disadvantage, accounting for factors such as income, education, employment, and housing quality. |
| Transfer status | Transferred from another hospital or rehabilitation unit to center of intervention |
| **Comorbidities** |  |
| Smoking status | Current, prior (quit ≥ 1 month ago), or never |
| Hypertension | Documented in history or recorded blood pressure > 130/80 on 3 or more occasions |
| Diabetes | Documented in history or receiving anti-hyperglycemic medications including insulin |
| Coronary artery disease | History of myocardial infarction, stable angina, or unstable angina |
| Congestive heart failure | Documented in history and severity classified based on the New York Heart Association (NYHA) heart failure classification |
| Chronic obstructive pulmonary disease | Documented in history and whether patient is not treated, on medications, or home oxygen |
| Dialysis | Patient is currently on hemodialysis or peritoneal dialysis |
| **Thrombotic risk factors** |  |
| Thrombophilia | Diagnosis of thrombophilia including antiphospholipid antibodies, excess Factor VIII, excess factor XI, Factor V Leiden mutation, hyperhomocysteinemia, Protein C deficiency, Protein S deficiency, prothrombin 20210A mutation, antithrombin deficiency, or other thrombophilia |
| Antiphospholipid antibodies | Diagnosis of antiphospholipid antibodies by a physician |
| Excess Factor VIII | Diagnosis of excess Factor VIII by a physician |
| Excess Factor XI | Diagnosis of excess Factor XI by a physician |
| Factor V Leiden mutation | Diagnosis of Factor V Leiden mutation by a physician |
| Hyperhomocysteinemia | Diagnosis of hyperhomocysteinemia by a physician |
| Protein C deficiency | Diagnosis of Protein C deficiency by a physician |
| Protein S deficiency | Diagnosis of Protein S deficiency by a physician |
| Prothrombin 20210A mutation | Diagnosis of Prothrombin 20210A mutation by a physician |
| Antithrombin deficiency | Diagnosis of antithrombin deficiency by a physician |
| Other thrombophilia | Diagnosis of another thrombophilia by a physician |
| Recent trauma within last 30 days | Patient suffered trauma within 30 days prior to receiving an inferior vena cava filter |
| Head | Patient suffered trauma to the head within 30 days prior to receiving an inferior vena cava filter |
| Long bones | Patient suffered trauma to the long bones within 30 days prior to receiving an inferior vena cava filter |
| Solid organ | Patient suffered trauma to a solid organ within 30 days prior to receiving an inferior vena cava filter |
| Spine | Patient suffered trauma to the spine within 30 days prior to receiving an inferior vena cava filter |
| Other trauma | Patient suffered trauma to another part of the body within 30 days prior to receiving an inferior vena cava filter |
| Prior major amputation | A prior below-knee, through-knee, or above-knee amputation |
| Prior venous thromboembolism | Prior diagnosis of venous thromboembolism with or without placement of an inferior vena cava filter |
| Family history of venous thromboembolism | History of venous thromboembolism in the patient’s family |
| Malignancy | Diagnosis of malignancy including cured or in remission or active malignancy |
| Pregnancy | Includes patients with a prior pregnancy who delivered more than 30 days ago or within the last 30 days and patients who were pregnant at the time of inferior vena cava filter placement |
| **Clinical presentation** |  |
| Pulmonary embolism | Diagnosis of pulmonary embolism by a physician categorized into the following: 1) asymptomatic (pulmonary embolism present but patient has no symptoms), 2) mild symptoms (shortness of breath, dizziness, fainting, cough, mild chest pain), 3) severe symptoms (pulse >110, systolic blood pressure <100mmHg, room air oxygen saturation <90%, severe chest pain, or severe shortness of breath), 4) massive pulmonary embolism (presents as impending respiratory arrest and/or right heart failure and is treated with immediate thrombolysis or thrombectomy), 5) chronic pulmonary embolism (prior remote pulmonary embolism that has resulted in chronic pulmonary hypertension necessitating treatment by surgical thrombectomy) |
| Lower extremity deep vein thrombosis | Diagnosis of deep vein thrombosis by a physician in the right leg, left leg, or both legs |
| Deep vein thrombosis location on the right leg | Location of deep vein thrombosis on the right leg if present, including the following veins: soleal/gastrocnemius, peroneal, tibial, popliteal, femoral, common femoral, common iliac, or inferior vena cava |
| Deep vein thrombosis location on the left leg | Location of deep vein thrombosis on the left leg if present, including the following veins: soleal/gastrocnemius, peroneal, tibial, popliteal, femoral, common femoral, common iliac, or inferior vena cava |
| Free floating thrombus | Free floating thrombus is defined as thrombus that extends more centrally than the primary deep vein thrombosis, typically a "tongue" of thrombus that is not attached to the vein wall. |
| Planned venous thrombolysis or thrombectomy | Patient is planned to receive catheter-based, pharmacologic, or operative venous thrombolysis or thrombectomy |
| Anticoagulation at therapeutic target | Options for this variable include: 1) Yes (patient is on anticoagulation at the time of filter insertion and has achieved therapeutic target. This includes patients in whom anticoagulation may have been held during the periprocedural period), 2) No, contraindicated (patient is not on anticoagulation due to a contraindication), and 3) No, unable to maintain therapeutic level (patient is on anticoagulation but has not achieved therapeutic levels as deemed by a physician) |
| Contraindications for anticoagulation | Patient has a contraindication for anticoagulation for 1 or more of the following reasons below |
| High risk of fall or injury | Patient deemed to be at high risk of fall or injury secondary to medical, psychological, or physical reasons by a physician |
| Heparin induced thrombocytopenia | Patient has laboratory diagnosed or suspected heparin induced thrombocytopenia |
| Non-bleeding complications (i.e., skin necrosis, allergy) | Patient has non-bleeding complications related to anticoagulation including skin necrosis and/or allergy |
| Planned major surgery | Patient has an upcoming planned major surgery |
| Recent cerebrovascular event (i.e., intracranial bleed or stroke) | Patient had a recent cerebrovascular event including intracranial bleed or stroke within 30 days of inferior vena cava filter placement |
| Recent major surgery | Patient had recent major surgery within 30 days of inferior vena cava filter placement |
| Recent trauma | Patient had a recent blunt or penetrating traumatic injury within 30 days of inferior vena cava filter placement |
| Recent or active bleeding | Patient has recent or active bleeding that, in the opinion of the treating physician, prevents the use of anticoagulation |
| Other | Patient has another contraindication for anticoagulation as deemed by the physician |
| Recurrent venous thromboembolism on anticoagulation | Patient has a recurrent venous thromboembolism while on therapeutic anticoagulation including a new or extension deep vein thrombosis (a new episode of acute deep vein thrombosis after a prior episode of deep vein thrombosis or additional acute thrombosis overlying an area of chronic thrombus after a prior deep vein thrombosis) or new pulmonary embolism |
| Major procedure planned | Patient has a major procedure planned within 30 days of inferior vena cava filter insertion, including bariatric, orthopedic, central nervous system, chest/abdomen, or other major surgery |
| Creatinine | Most recent pre-operative creatinine within 6 months prior to surgery (umol/L) |
| **Anatomic and inferior vena cava filter characteristics** |  |
| Planned duration of inferior vena cava filter | Options include 1) temporary (the filter was placed with the primary intent to remove it later; note that the decision is not binding and a planned temporary filter could eventually become a permanent filter if it is left in), 2) permanent (the filter was placed with the primary intent of not removing it; note that this choice is not binding and a permanent filter may become temporary if decided to be removed later), or 3) not reported |
| Inferior vena cava filter placement location | Location where the procedure took place, including the fluoroscopy suite, bedside, or not reported |
| Access vein | Vein used to obtain access to place inferior vena cava filter including right jugular, left jugular, right femoral, left femoral, right leg non-femoral vein, left leg non-femoral vein, or not reported |
| Landing site | The landing site is defined as the vein where the filter hooks attach to the vein wall, including 1) infrarenal (entire filter below the lowest renal vein), 2) pararenal (apex of filter at or above the lowest renal vein, but filter anchored below the lowest renal vein), 3) suprarenal (filter anchored above lowest renal vein), 4) right iliac vein, 5) left iliac vein, or 6) bilateral iliac veins |
| Imaging available to place filter | Imaging equipment available to place inferior vena cava filter, including fluoroscopy, transcutaneous ultrasound, intravascular ultrasound, or not reported |
| Abnormal venous anatomy | Any abnormal or aberrant venous anatomy that affected the decision of where to place the filter or the type of filter used, including inferior vena cava compression, tortuosity, or duplication, accessory renal vein, or large low lying gonadal vein |
| **Functional status** |  |
| Living status | Last living status prior to hospitalization (home, nursing home, homeless, or not reported) |
| Ambulatory status | Pre-operative ambulatory status, including 1) ambulatory independently with or without prosthesis, 2) ambulatory with assistance (e.g., cane, walker, or person), 3) wheelchair-dependent, 4) bedridden (full assist to get to chair), or 5) not reported |
| **Medications** |  |
| Acetylsalicylic acid | Includes drugs that contain acetylsalicylic acid taken within 36 hours of the procedure |
| P2Y12 antagonist | Includes clopidogrel, prasugrel, ticlopidine, and ticagrelor taken within 36 hours of the procedure |
| Statin | Includes atorvastatin, fluvastatin, lovastatin, pitavastatin, pravastatin, rosuvastatin, simvastatin, or a combination medication including a statin taken within 36 hours of the procedure |
| Oral anticoagulant | Includes warfarin, dabigatran, rivaroxaban, or other oral anticoagulant taken on the same calendar day as the procedure |
| Intravenous or subcutaneous anticoagulant | Includes intravenous or subcutaneous unfractionated heparin, low molecular weight heparin, fondaparinux, argatroban, bivalirudin, desirudin, lepirudin, or other intravenous or subcutaneous anticoagulant taken on the same calendar day as the procedure |
| Estrogen-containing therapy | Includes oral contraceptives or other estrogen-containing therapies taken by the patient prior to the procedure |

**Supplementary Table 2. Selection of Extreme Gradient Boosting (XGBoost) model hyperparameters using grid search and cross validation**

| **Hyperparameter** | **Values tested through grid search and cross validation*** | **Optimal value chosen to maximize AUROC** |
| --- | --- | --- |
| Number of rounds | 50, 100, 150, 200, 250, 300, 350, 400, 450, 500 | 200 |
| Maximum tree depth | 2, 3, 4, 5, 6, 7, 8, 9 | 3 |
| Learning rate | 0.4, 0.3, 0.2, 0.1, 0.05, 0.01, 0.001 | 0.01 |
| Gamma | 0, 0.1, 1, 1.5, 2 | 0 |
| Column sample by tree | 0.5, 0.6, 0.7, 0.8, 0.9, 1 | 1 |
| Minimum child weight | 1, 3, 5, 7, 10 | 1 |
| Subsample | 0.5, 0.6, 0.7, 0.8, 0.9, 1 | 0.9 |

*Grid search and cross validation are exhaustive methods that iteratively train and evaluate models using every combination of specified hyperparameter values and selects the set of hyperparameter values that optimize model performance.

Abbreviation: AUROC (area under the receiver operating characteristic curve).


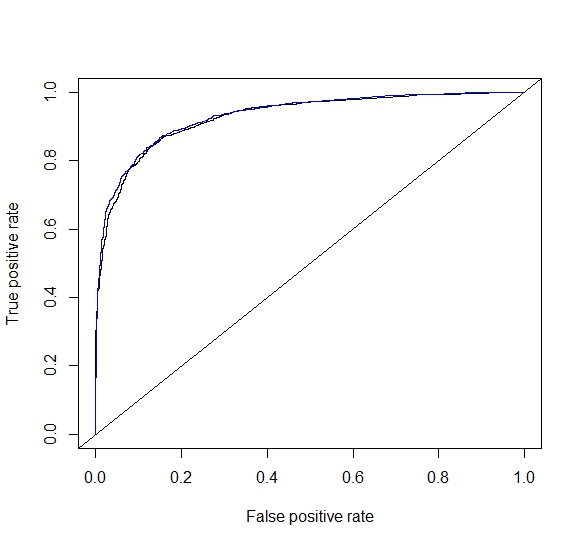


AUROC (95% CI)

Age < 60: 0.93 (0.92 – 0.94)

Age ≥ 60: 0.93 (0.92 – 0.94)

**Supplementary Figure 1. Receiver operating characteristic curve for predicting 1-year filter-related complications following inferior vena cava filter placement using Extreme Gradient Boosting (XGBoost) model with subgroup analysis based on age.** AUROC (area under the receiver operating characteristic curve), CI (confidence interval).


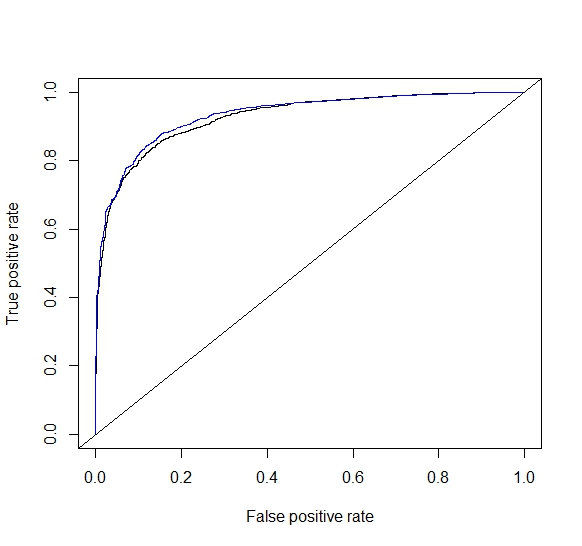


AUROC (95% CI)

Male: 0.93 (0.92 – 0.94)

Female: 0.93 (0.92 – 0.94)

**Supplementary Figure 2. Receiver operating characteristic curve for predicting 1-year filter-related complications following inferior vena cava filter placement using Extreme Gradient Boosting (XGBoost) model with subgroup analysis based on sex.** AUROC (area under the receiver operating characteristic curve), CI (confidence interval).


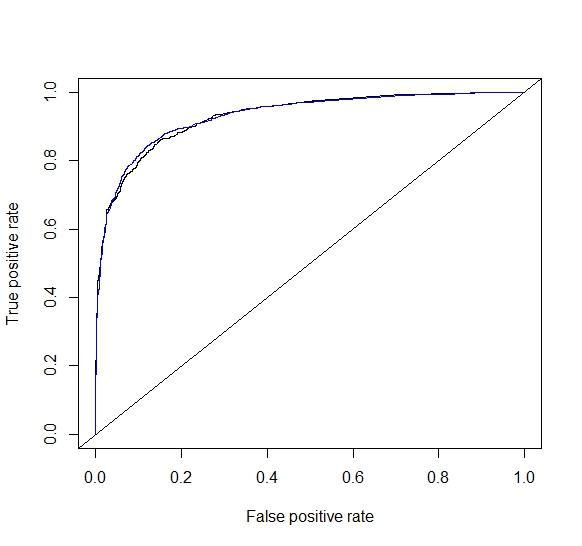


AUROC (95% CI)

White: 0.93 (0.92 – 0.94)

Non-White: 0.93 (0.92 – 0.94)

**Supplementary Figure 3. Receiver operating characteristic curve for predicting 1-year filter-related complications following inferior vena cava filter placement using Extreme Gradient Boosting (XGBoost) model with subgroup analysis based on race.** AUROC (area under the receiver operating characteristic curve), CI (confidence interval).


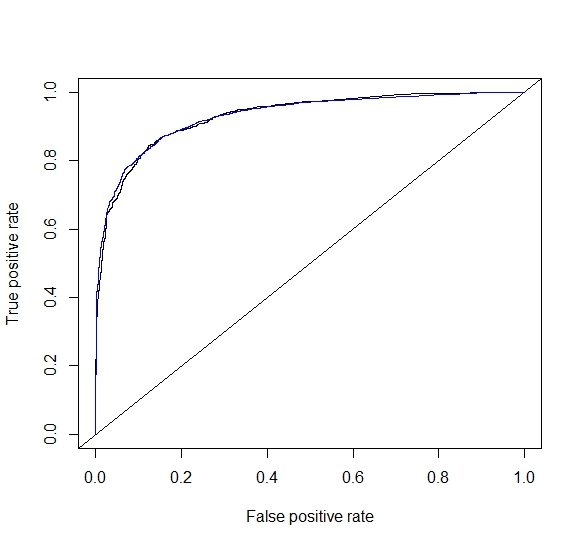


AUROC (95% CI)

Hispanic: 0.93 (0.92 – 0.94)

Non-Hispanic: 0.93 (0.92 – 0.94)

**Supplementary Figure 4. Receiver operating characteristic curve for predicting 1-year filter-related complications following inferior vena cava filter placement using Extreme Gradient Boosting (XGBoost) model with subgroup analysis based on ethnicity.** AUROC (area under the receiver operating characteristic curve), CI (confidence interval).


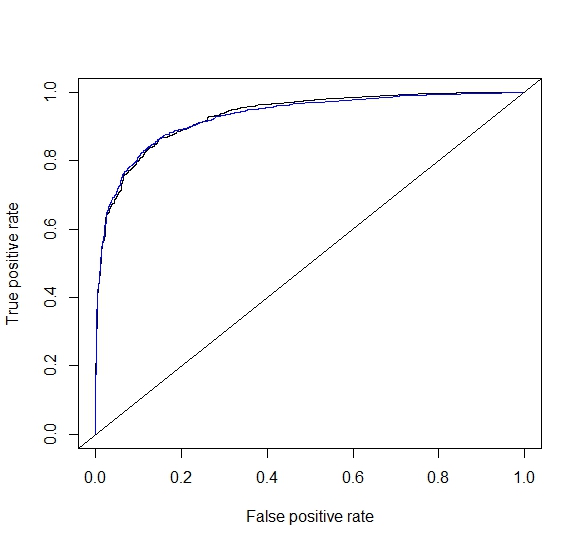


AUROC (95% CI)

Rural: 0.93 (0.92 – 0.94)

Non-rural: 0.93 (0.92 – 0.94)

**Supplementary Figure 5. Receiver operating characteristic curve for predicting 1-year filter-related complications following inferior vena cava filter placement using Extreme Gradient Boosting (XGBoost) model with subgroup analysis based on rurality of residence.** AUROC (area under the receiver operating characteristic curve), CI (confidence interval).


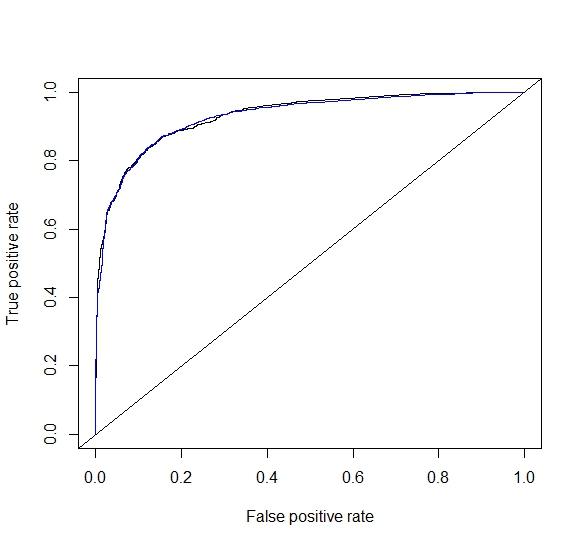


AUROC (95% CI)

ADI ≥ 50%: 0.93 (0.92 – 0.94)

ADI < 50%: 0.93 (0.92 – 0.94)

**Supplementary Figure 6. Receiver operating characteristic curve for predicting 1-year filter-related complications following inferior vena cava filter placement using Extreme Gradient Boosting (XGBoost) model with subgroup analysis based on median Area Deprivation Index (ADI) percentile.** AUROC (area under the receiver operating characteristic curve), CI (confidence interval).


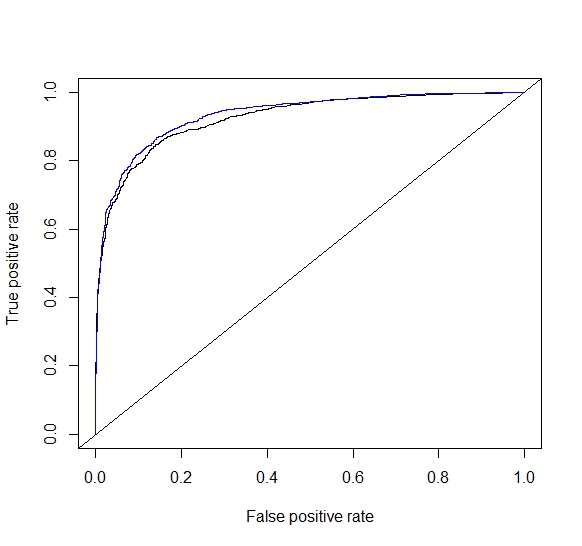


AUROC (95% CI)

Temporary: 0.93 (0.92 – 0.94)

Permanent: 0.94 (0.93 – 0.95)

**Supplementary Figure 7. Receiver operating characteristic curve for predicting 1-year filter-related complications following inferior vena cava filter placement using Extreme Gradient Boosting (XGBoost) model with subgroup analysis based on planned duration of filter.** AUROC (area under the receiver operating characteristic curve), CI (confidence interval).


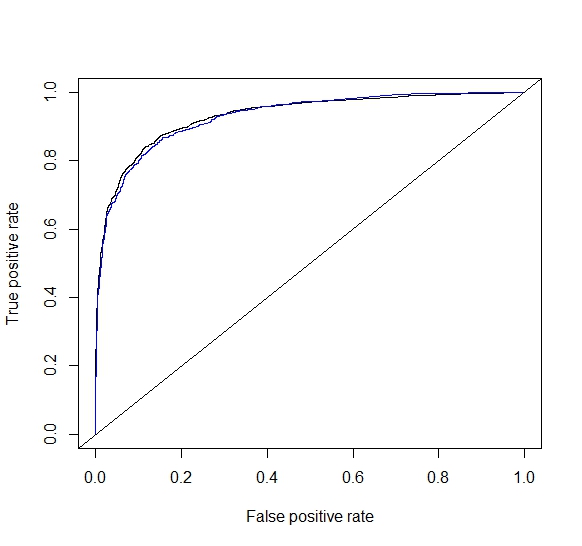


AUROC (95% CI)

Infrarenal: 0.93 (0.92 – 0.94)

Non-infrarenal: 0.93 (0.92 – 0.94)

**Supplementary Figure 8. Receiver operating characteristic curve for predicting 1-year filter-related complications following inferior vena cava filter placement using Extreme Gradient Boosting (XGBoost) model with subgroup analysis based on landing site of filter.** AUROC (area under the receiver operating characteristic curve), CI (confidence interval).


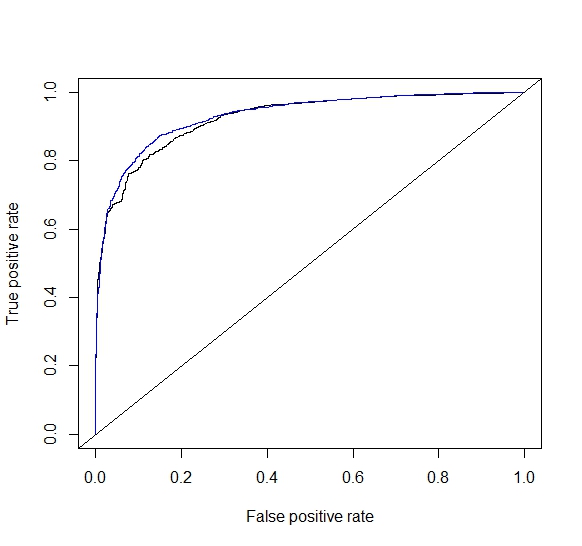


AUROC (95% CI)

Prior IVC filter: 0.92 (0.91 – 0.94)

No prior IVC filter: 0.93 (0.92 – 0.94)

**Supplementary Figure 9. Receiver operating characteristic curve for predicting 1-year filter-related complications following inferior vena cava (IVC) filter placement using Extreme Gradient Boosting (XGBoost) model with subgroup analysis based on presence of prior IVC filter placement.** IVC (inferior vena cava), AUROC (area under the receiver operating characteristic curve), CI (confidence interval).
